# Reproducibility of exhaled nitric oxide in smokers and non-smokers: relevance for longitudinal studies

**DOI:** 10.1186/1471-2466-8-4

**Published:** 2008-02-28

**Authors:** Abraham Bohadana, Jean-Pierre Michaely, Dan Teculescu, Pascal Wild

**Affiliations:** 1 Institut National de la Santé et de la Recherche Médicale, INSERM, ERI 11, 54505 Vandoeuvre – lès-Nancy, France; 2 Service de Pneumologie, Centre Hospitalier Universitaire de Nancy, 54511 Vandoeuvre-lès-Nancy, France; 3 Département Epidémiologie en Entreprises, INRS, 54511 Vandoeuvre-lès-Nancy, France

## Abstract

**Background:**

Currently, there is much interest in measuring fractional exhaled nitric oxide (**FE_NO_**) in populations. We evaluated the reproducibility of **FE_NO _**in healthy subjects and determined the number of subjects necessary to carry out a longitudinal survey of **FE_NO _**in a population containing smokers and non-smokers, based on the assessed reproducibility.

**Methods:**

The reproducibility of **FE_NO _**was examined in 18 healthy smokers and 21 non-smokers. **FE_NO _**was assessed once at 9 AM on five consecutive days; in the last day this measurement was repeated at 2 PM. Respiratory symptoms and medical history were assessed by questionnaire. The within- and between-session repeatability of **FE_NO _**and log-transformed **FE_NO _**was described. The power of a longitudinal study based on a relative increase in **FE_NO _**was estimated using a bilateral t-test of the log-transformed **FE_NO _**using the between-session variance of the assay.

**Results:**

**FE_NO _**measurements were highly reproducible throughout the study. **FE_NO _**was significantly higher in males than females regardless of smoking status. **FE_NO _**was positively associated with height (p < 0.001), gender (p < 0.034), smoking (p < 0.0001) and percent FEV_1_/FVC (p < 0.001) but not with age (p = 0.987). The between-session standard deviation was roughly constant on the log scale. Assuming the between-session standard deviation is equal to its longitudinal equivalent, either 111 or 29 subjects would be necessary to achieve an 80% power in detecting a 3% or a 10% increase in **FE_NO _**respectively.

**Conclusion:**

The good reproducibility of **FE_NO _**is not influenced by gender or smoking habits. In a well controlled, longitudinal study it should allow detecting even small increases in **FE_NO _**with a reasonable population size.

## Background

Fractional exhaled nitric oxide (**FE_NO_**) is now widely used as a surrogate marker for eosinophilic airway inflammation [1]. Yet, several personal and environmental factors including active or passive smoking may influence **FE_NO _**thus acting as causes of bias [2].

Good reproducibility is an important precondition for using a physiological test both for clinical and epidemiological purposes [3]. While the variability and reproducibility of **FE_NO _**have been evaluated in patients with asthma, studies in healthy subjects usually excluded smokers [4-7]. Since exposure to cigarette smoke decreases **FE_NO _**[8-13] a given instrumental or biological variation will yield higher coefficients of variation if the denominator is lower.

Currently, there is much interest in assessing longitudinal changes in **FE_NO _**levels in populations. Ensuring a low variability and good reproducibility in this setting is critical in distinguishing true biological changes from other sources of variation. In this study, we examined the influence of smoking status and gender on the reproducibility of FE_NO _measurements in a group of healthy individuals. Then, based on the reproducibility, we determined the sample size necessary to carry out a longitudinal study on the evolution of **FE_NO_**, a matter on which there is virtually no information in the literature.

## Methods

### Subjects and Protocol

Thirty-nine young subjects were recruited among clerks, technicians, engineers, postgraduate students and medical residents from the University of Nancy. There were 21 non-smokers (10 males and 11 females) and 18 smokers (9 males and 9 females). The selection criteria were a negative history of respiratory symptoms and allergic diseases (e.g. rhinitis, eczema), and normal spirometry. Subjects with acute respiratory infection in the last 4 weeks were excluded. None of the volunteers was taking any drug or medication. All subjects gave informed consent and the research protocol was approved by our local ethics in medical research committee.

Subjects were approached a first day when a brief respiratory symptoms questionnaire was administered through a face-to-face interview and pulmonary function tests were performed. The questionnaire covered personal and demographic information, past and present chest diseases, respiratory symptoms and allergies, and past and present smoking habits. After inclusion, six sessions of three measurements were planned over one week. All subjects had their **FE_NO _**measured once on five consecutive days, from Monday through Friday, between 09:00–10:00 h during visits 1, 2, 3 and 4; during the last visit **FE_NO _**was measured twice between 09:00–10:00 h and 14:00–15:00 h.

Spirometry was carried out in the sitting position, using an electronic spirometer (Oscilolink, Datalink, Montepellier, France). Forced vital capacity (FVC) and forced expiratory volume in one second (FEV_1_) were obtained by having the subject expire forcefully after a maximal inspiratory maneuver. At least three forced expiratory maneuvers, satisfactory according to recommended criteria [14] were recorded. The curve with the highest sum of FVC+ FEV_1 _was used for analysis. Curves with an FEV_1_/FVC ratio of 80% or higher *and *a predicted [15] FEV_1 _≥ 75% were considered as normal.

**FE_NO _**was measured according to the ATS/ERS recommendations [16]. Measurements were done using a chemiluminescence analyzer (NIOX^® ^2.0 system; Aerocrine AB, Solna, Sweden). The subject was in the sitting position and exhaled against an oral pressure of 5 cm H_2_0 – sufficient to close the velum – at a flow rate of 50 mL/s. At each session, three correctly performed exhalations were recorded. Any exhalation which did not meet the ATS/ERS requirements was rejected by the NIOX system and the subject was asked to perform a new exhalation maneuver. Calibrations were performed every 13 days according to the standards of the manufacturer. Subjects avoided eating for 1 hour and smoking for 8 hours before testing.

### Statistical analysis

Statistical analysis was performed using the Stata package [17]. The Shapiro-Wilks test was used to check the normality and log-normality of the measurements. Reproducibility of **FE_NO _**measurements was assessed by the Bland Altman analysis [18]. Within-session repeatability was assessed by plotting the difference between each of the three measurements of a session and their session mean against the session mean. This procedure generated three data points by subject by session, thus yielding 702 data points (39 subjects × 3 measures by session × 6 sessions = 702). Between-session repeatability was calculated by plotting the difference between a mean session FE_NO _and the overall individual mean against the overall individual mean both on the natural and log-transformed (on base 10) basis. This procedure generated 1 data point by subject by session thus yielding 234 data points (39 subjects × 6 sessions = 234). The possible learning effect was assessed by comparing the 6 successive sessions and the morning sessions on day 1 and day 5 using a repeated measure analysis of variance. In turn, the diurnal variation was assessed by comparing, using a matched t-test, the morning session vs. the afternoon session on day 5.

A multiple linear regression model was used to assess the association between log-transformed **FE_NO_**, taken as the dependent variable, and other, independent variables including age, height, sex, smoking habits and spirometric variables.

A sample size determination was calculated to achieve an 80% power in a study in which **FE_NO _**would be measured twice e.g. before and after exposure to some risk factor. The statistical test is based on the individual difference of log-transformed values at time 2 and time 1 tested to be equal to zero by a standard two-sided Student test. In other words, it tests an increase in percent of the initial measurement. The expected standard deviation of this difference of (log-transformed) measurements is estimated using the within-subject between-session standard deviation of the log-transformed mean of the three measurements which is assumed to be equal to the individual long-term variability.

## Results

**FE_NO _**levels were measured on 702 occasions. The normality of the data was rejected (Shapiro-Wilk test, p < 0.0001), mainly because of 1 outlying subject, but not the log-normality. Descriptive statistics by sex and smoking habits are presented in Table 1. Smoking men were slightly older and had greater cigarette consumption than smoking women but the differences were not significant (p values of 0.302 and 0.360, respectively). Among non-smokers, women were older than men (p = 0.003). BMI was within the normal range and comparable among the four groups.

**Table 1:** Baseline characteristics of participants. Values are mean (SD).

**Parameter**	**Smokers**		**Non-Smokers**	
	
	**Men**	**Women**	**Men**	**Women**
**Nb of subjects**	9	9	10	11
**Age, years**	34.6 (9.9)	29.7 (9.6)	26.2 (2.0)	38.5 (11.3)
**Height, cm**	178.2 (8.4)	166.8 (3.2)	176.4 (7.0)	166.1 (5.4)
**Weight, kg**	73.8 (8.3)	64.6 (12.5)	73.8 (9.3)	63.2 (9.0)
**BMI, kg/m^2^**	23.2 (2.0)	23.2 (4.7)	23.7 (2.6)	22.9 (2.7)
**Cigarettes, p.y**	8.8 (12.1)	4.6 (5.7)	0	0
**Years smoking**	14.2 (9.6)	8.5 (5.1)	-	-
**FEV1**				
**L**	3.86 (0.69)	2.93 (0.32)	3.97 (0.55)	2.83 (0.49)
**% predicted**	92.2% (11.1)	90.3% (7.4)	91.5% (10.5)	94.4% (8.9)
**FVC**				
**L**	4.84 (0.81)	3.71 (0.48)	4.97 (0.63)	3.53 (0.58)
**% predicted**	96.2% (9.6)	99.8% (11.6)	96.8% (6.1)	101.6% (9.7)
**FEV1/FVC**				
**% observed**	79.7(5.1)	79.2 (5.6)	80.1 (8.8)	80.4% (5.9)

Abbreviations:

BMI: Body mass index

p.y = pack years

FEV1: Forced expiratory volume in one second

FVC: Forced vital capacity

The overall within-session standard deviation was 1.78 parts per billion (ppb); males and non-smokers displayed higher **FE_NO _**levels and correspondingly higher standard deviations. **FE_NO _**values at each visit are presented by gender and smoking status in Table 2. The geometric mean **FE_NO _**for male smokers (average = 15.8 ± 1.66) was 1.37 times higher than in female smokers (average = 11.5 ± 1.82). The mean FENO for male non-smokers (average = 33.1 ± 2.4) was 2.04 times higher than in female non-smokers (average = 16.2 ± 1.51). Overall, **FE_NO _**measurements made at different visits were highly reproducible throughout the study. There was no significant day-to-day variation. Also, morning and afternoon values at visit 5 were close to each other showing no significant diurnal variation. Finally, the repeated measure ANOVA shows that **FE_NO _**varies significantly over the week with a decrease of about 12% between the two first measurements and the others. However, it can be seen that among non-smoking females this decrease is not constant. No diurnal difference was noted comparing the last 2 sessions.

**Table 2:** Geometrical mean (ppb) and geometrical SD of FE_NO _measurement on six occasions in smokers and non-smokers stratified by gender.

**Subjects**		**Visit 1**	**Visit 2**	**Visit 3**	**Visit 4**	**Visit 5a**	**Visit 5b**	**Total**
**Smokers**	**Men (n = 9)**	17.8 (1.82)	15.8 (1.78)	15.1 (1.58)	15.8 (1.66)	14.8 (1.62)	16.2 (1.74)	**15.8 (1.66)**
	**Women (n = 9)**	12.6 (2.00)	13.2 (2.09)	11.7 (1.78)	11.0 (1.82)	10.5 (1.86)	10.0 (1.62)	**11.5 (1.82)**
**Non-smokers**	**Men (n = 10)**	33.9 (2.57)	36.3 (2.45)	30.9 (2.57)	32.4 (2.51)	32.4 (2.51)	32.4 (2.51)	**33.1 (2.40)**
	**Women (n = 10)**	17.4 (1.78)	16.2 (1.51)	14.4 (1.51)	17.4 (1.41)	16.6 (1.51)	15.8 (1.48)	**16.2 (1.51)**

Visit 5a: 9:00 – 10:00 h

Visit 5b: 14:00 – 15:00 h.

Table 3 displays the coefficients of the multiple linear regression model used to assess the association between **FE_NO _**and several independent variables. Gender, smoking, height and the FEV_1_/FVC ratio expressed in percent predicted, but not age, were significantly associated with **FE_NO_**. The estimated difference of -0.098 on the decimal log scale between men and women, adjusted on the other factors, corresponds to a predicted **FE_NO _**value for men which is multiplied by 10^0.098 ^= 1.25 or equivalently an **FE_NO _**value among women which is 25% lower than among men. Similarly the **FE_NO _**among non-smokers is 69% larger than among smokers and it increases by 2.6% by centimetre height and by 4% by %FEV_1_/FVC. The influence of age is negligible in this model and is far from statistical significance in any of the models considered. It is to be noted (data not shown) that height was non-significant when the FEV_1_/FVC ratio was replaced by the FEV_1 _or if the spirometric variables were omitted from the model. When performing unadjusted univariate analyses, the coefficients for smoking, age, height and FEV_1_/FVC were very similar to the coefficients from the multiple model and were all (except age) statistically significant. The unadjusted gender difference (62%) is however much larger than the adjusted one.

**Table 3:** Multiple linear regression model with log-FE_NO _as the dependent variable in relation to age, height, gender, smoking status and spirometry among 39 healthy subjects.

**Variable**	Coefficient	Std. Error	t	p	[95% confidence interval]	
**Constant**	-2.13	0.641	-3.32	0.001	-3.394	-0.867
**Age (year)**	0.00003	0.002	-0.02	0.987	-0.003	0.003
**Height (cm)**	0.011	0.003	3.71	<0.001	0.005	0.017
**Gender: Men vs. Women**	-0.098	0.046	-2.14	0.034	-0.188	-0.008
**Smoker vs. Non-smoker**	-0.227	0.033	-6.94	<0.001	-0.292	-0.163
**FEV_1_/FVC (% predicted)**	0.017	0.002	7.40	<0.001	0.013	0.022

**Figure 1:**
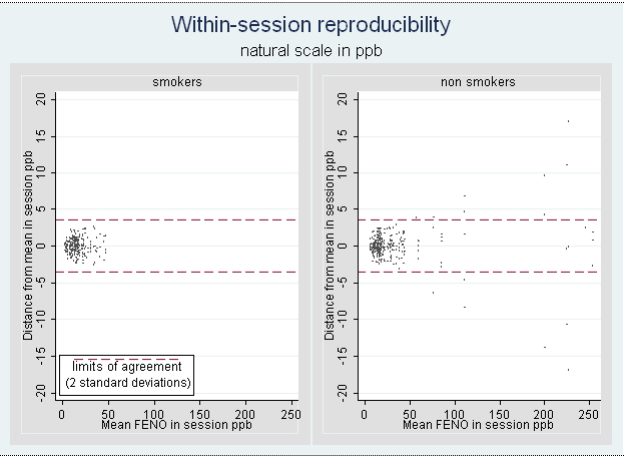
Within-session repeatability of **FE_NO _**in smoking groups.

Bland-Altman analysis of distance from session means using all 702 measurements yielded limits of agreement of 3.56 ppb (i.e. 2 × SD or 1.78 × 2 = 3.56). Figure 1 shows the within session repeatability of **FE_NO _**for all recorded measurements separately for smokers and non-smokers. These figures show a variability which is similar among smokers and non-smokers, for any given mean **FE_NO _**value.

**Figure 2:**
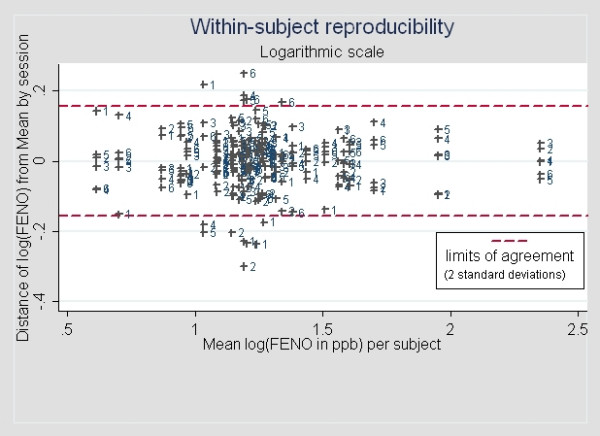
Between-session repeatability of (log-transformed) **FE_NO _**as a function of subject's mean **FE_NO _**level. The numbers correspond to the session number (from 1 to 6).

In a longitudinal, long-term follow up study, only three measurements will be taken at each time point corresponding to a measurement session in the present series of data so the individual parameters of interest are the mean of the three measurements and its variance. This variance can be estimated from our trial by computing the variance of the 6 sessions. To compensate for the phenomenon of heteroskedasticity (i.e; the variance increases with the mean) – documented by a 0.89 correlation coefficient between the subject-specific between session standard deviation and mean – we log-transformed **FE_NO _**values, yielding the log **FE_NO _**(Figure 2). The variability is much less dependent on the mean, the correlation coefficient between mean and standard deviation being now – 0.29.

**Figure 3:**
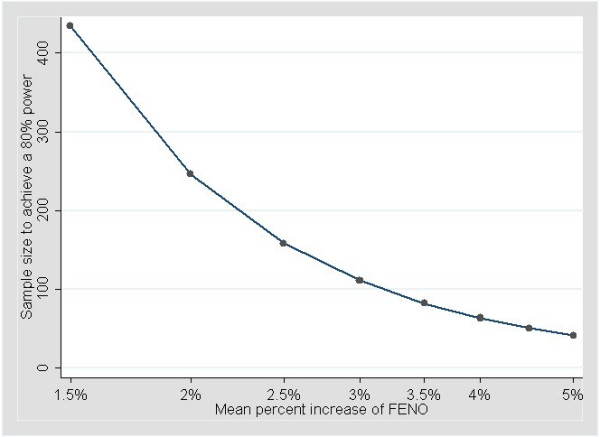
Number of subjects necessary to achieve an 80% power to detect a given increase in percent of the initially measured **FE_NO _**level in a second measure.

Figure 3 represents the number of subjects necessary to achieve an 80% power to detect a given (multiplicative) increase in percent of the initially measured **FE_NO _**level in a second measure. The power calculations are based on an estimated between-session within-subject standard deviation of 0.078 corresponding to a geometric standard deviation of 1.081. To achieve an 80% power to detect a mean 3% increase in exhaled NO, 111 subjects have to be included. For a more clinically significant, say, 10% mean increase, this number drops to 29.

## Discussion

In this study, we demonstrate that **FE_NO _**measurements are reproducible, a finding consistent with results reported previously in healthy subjects and patients with asthma [4-7]. Yet, we provide new knowledge by showing that the reproducibility of **FE_NO _**is not influenced by gender nor adversely affected by smoking status in smokers refraining from smoking before testing. In addition, we show that significant changes in **FE_NO _**are potentially detectable with fairly small numbers of subjects, an aspect of practical importance when planning epidemiological studies. However, it should be pointed out that our calculations were based on the assumption that the variance of the mean **FE_NO _**measurement within the week in which the essay was conducted represents the intrinsic long-term variability of **FE_NO _**in absence of any external factor influencing it. Although this hypothesis seems reasonable – as supported by the remarkable seasonal stability of **FE_NO _**[7] – only a much longer follow up of this measurement in a similar population could validate it. Finally, it should be stressed that this reasoning does not apply to managing individual asthmatics in the clinical setting.

Our study differed from those quoted above [4-7] in three ways. First, we examined males and females separately, while some authors preferred to examine males only [5] or merge males and females together [6,7]; one study did not mention the subjects' gender [4]. Second, we examined smokers and non-smokers of both sexes separately, while the above mentioned studies [4-6] did not take smoking status into account. Finally, our series included only healthy subjects while other series included asthmatics as well [5,6].

For the sake of comparability, we used a protocol similar to that of Kharitonov and colleagues [6]. The within-session standard deviation was close to that noted by these authors (1.8 ppb vs. 2.1 ppb) although it increased with the mean **FE_NO_**. However, in contrast to Kharitonov et al, we did not exclude any *a priori *outliers as it would lead to removing almost all measurements of subjects with high **FE_NO _**values. If such procedure were applied in any clinical or epidemiological study it would imply removing the most interesting subjects.

By plotting the distance between the session means and the overall subject specific mean vs. the latter we showed the within-subject between-session variance increased with the subject mean; this feature disappeared when using log-transformed measurements, which we would therefore recommend as the relevant variable. This means simply that any evolution of a subject's **FE_NO _**is better expressed in percent of the initial value rather than as an increase or decrease in the absolute value.

The relationship between gender and **FE_NO _**has been examined previously. Olin and colleagues [19] measured **FE_NO _**in a large random adult general population sample (n = 2,200) and found that when both height and gender were included in multiple regression model the contribution of gender was not significant. They postulated that the alleged association **FE_NO_**/male gender was probably due to a height-dependent increase in the total airway mucosal surface area that produces NO. However, conflicting results were published by others. Olivieri and colleagues [20] measured **FE_NO _**in 204 healthy, non-smoking, non-atopic individuals and documented significantly higher **FE_NO _**values in men compared with women. Travers and colleagues [21] examined subjects aged between 25 and 75 years and found that sex, atopy and smoking status affected **FE_NO _**levels; they presented reference ranges adjusted for these factors. More recently, Robin Taylor and colleagues [22] examined 895 adults aged 32 and found that **FE_NO _**levels were nearly 25% less in females and suggested that reference values should be stratified by sex. In the present study we also found that women had significantly lower **FE_NO _**than men and that the association between **FE_NO _**and gender remained significant when height was included in the regression model.

There is evidence that genetic factors might play a role on the genesis of the reported gender-related differences in **FE_NO_**. Graserman and colleagues [23] studied NO and genetic variants in NO synthases in 105 healthy non-smoking and smoking subjects. They found **FE_NO _**to be significantly higher in males than females among both non-smokers and smokers. More importantly, they noted that healthy non-smoking females with greater number of repeats in neuronal NO synthase had significantly lower NO levels than did females with fewer numbers of repeats. They concluded that variants in the neuronal NO synthase gene contribute to the variability of airway NO concentrations in healthy females. More recently, Lund and colleagues [24] examined 377 adult twins identified through the Norwegian Twin Registry and showed that genetic effects accounted for 60% of the variation in **FE_NO_**. The influence of genetic factors on our results is not known and clearly more studies are necessary to clarify this issue.

Our study confirms the well-established association between cigarette smoking and **FE_NO _**levels [8-13]. The mechanisms by which smoking reduces **FE_NO _**are not completely understood. Several possibilities have been proposed including *(i) *a reduction of the endogenous NO synthesis by feedback inhibition due to the high concentrations of NO contained in the cigarette smoke [9]*(ii) *an inadequate supply of cofactors necessary for NO production [25] and *(iii) *an increase in the breakdown of NO [26]. Recently, Malinovschi and colleagues [10] examined the effect of past, current and passive smoking on **FE_NO _**in a general population sample using flow-independent NO exchange parameters. They found lower **FE_NO _**levels in current smokers and attributed this to reduced NO levels in both the airways and alveoli. Conversely, Pietropaoli and colleagues [27] measured **FE_NO _**at different expiratory flow rates in asymptomatic smokers and age-matched non-smokers and concluded that the diminished NO expired by smokers resulted from diminished NO production by the tissues of the conducting airways and not by the alveoli. Whatever the mechanism, there appears that **FE_NO _**levels decrease with increasing years smoked and with recent smoking [13]. Had it been present in our study, the latter factor could have produced variations in **FE_NO _**levels and adversely affected our reproducibility. However, to prevent such possibility and achieve stability we asked our subject to avoid smoking for 8 hours before **FE_NO _**measurements.

Atopy might have influenced baseline **FE_NO _**levels of our subjects [19,28,29] although we are not aware of such an effect in absence of any airway inflammation. However, while the NO level of atopic subjects can be expected to increase specifically under the influence of a risk factor [30], in absence of any such factor, there is no reason it should vary considerably. Excluding atopic subjects in a longitudinal study would thus bias the results of that study towards the absence of effect. By consequence, we decided not to screen our subjects for asymptomatic atopy (e.g. by performing skin prick tests to common allergens) nor eliminate outliers.

A final word must be said about our protocol. As with other tests, **FE_NO _**measurement may be influenced by technical and biological sources of variation. To deal with the former, all measurements were carried out by the same operator, using the same instrument – which met the ATS criteria for precision and accuracy [14] – according to a standardized procedure. Further, care was taken to ensure the subjects' comprehension, comfort and cooperation during the procedure. As for biological sources, we took care to record carefully the subjects' recent exposures and activities. In addition, we dealt with the possible role of circadian rhythms by asking each subject to come to the laboratory every day at the same hour. Incidentally, our average **FE_NO _**values were within the limits of reported reference values established using NO analyzers of the same manufacturer [21,31]. For smokers, our values are below both the upper limit of 36.4 ppb for females and the upper limit of 45.9 ppb for males proposed by Travers and colleagues [21]. For non-smokers, our values are similar to the 31.2 ppb value predicted for men and below the 29.7 ppb value predicted for women by Olin and colleagues [31] and far below the upper limit of 44.6 and 56.5 ppb proposed respectively for women and men by Travers and colleagues [21].

## Conclusion

In summary, this study improves our understanding of the reproducibility of **FE_NO _**in two ways. First, it shows that this reproducibility is not influenced by gender or smoking status in subjects refraining from smoking before **FE_NO _**assessment. Second, it provides evidence that **FE_NO _**is potentially useful in the survey of populations, fairly small samples being necessary to assess significant changes in **FE_NO _**levels. This finding could be of practical importance for the survey of populations at risk of asthma since traditional means of monitoring such as tests of lung function and bronchial provocation tests are not directly related to airway inflammation. Further studies, carried out over longer periods of time, would be necessary to confirm the assumption that the short-term variance of **FE_NO _**represents its intrinsic long-term variability.

## Abbreviations

FE_NO_: Fractional exhaled nitric oxide; FEV_1_: Forced expiratory volume in one second; FVC: Forced vital capacity.

## Competing interests

The author(s) declare that they have no competing interests.

## Authors' contributions

AB: study conception and design, sample collection, data analysis and interpretation; involved in drafting the article. J-P M: data acquisition, analysis and interpretation. DT: data acquisition, analysis and interpretation, critically reviewing the draft for important intellectual content. PW: study conception and design, data interpretation and statistical analysis; involved in drafting the article.

## Pre-publication history

The pre-publication history for this paper can be accessed here:


